# REMINISCENCE AND DIGITAL STORYTELLING TO IMPROVE WELL-BEING OF OLDER ADULTS WITH COGNITIVE IMPAIRMENT

**DOI:** 10.1093/geroni/igac059.041

**Published:** 2022-12-20

**Authors:** Ling Xu, Noelle Fields, Kathryn Daniel, Brooke Troutman, Daisha Cipher

**Affiliations:** University of Texas at Arlington, Arlington, Texas, United States; The University of Texas at Arlington, Arlington, Texas, United States; University of Texas at Arlington, Arlington, Texas, United States; Air Force Academy, Air Force Academy, Colorado, United States; The University of Texas at Arlington, Arlington, Texas, United States

## Abstract

To address the growing concern about loneliness and diminished well-being among persons with cognitive impairment, an intergenerational intervention based on reminiscence and digital storytelling was offered by trained college student volunteers to older adults living in the community. A randomized controlled trial was used to assess the effects of the intervention. Younger and older adult participants were randomly paired and assigned to reminiscence (n=20) or control (social wellness, n=16) groups. Data were collected at baseline, mid-intervention, and at the end of the intervention. Friedman tests for non-normally distributed outcome variables and one-way repeated measures ANOVA for normally distributed outcome variables were conducted. Results showed that emotional loneliness, total social and emotional loneliness, quality of life, and positive affect significantly improved among older adults in the reminiscence group, especially between baseline and posttest. Results suggest that weekly intergenerational engagements with young adults benefit older adults with psychological well-being.

